# Children’s Perceived Competence Scale: reevaluation in a population of Japanese elementary and junior high school students

**DOI:** 10.1186/s13034-018-0241-4

**Published:** 2018-07-11

**Authors:** Yukiyo Nagai, Kayo Nomura, Masako Nagata, Tetsuji Kaneko, Osamu Uemura

**Affiliations:** 1 Department of Pediatrics, Japanese Red Cross Nagoya Daini Hospital, 2-9 Myoken-cho Showa-ku, Nagoya, Aichi 466-8650 Japan; 2 Graduate School of Education and Human Development, Nagoya University, Furo-cho Chikusa-ku, Nagoya, Aichi 464-8601 Japan; 3 Department of Clinical Trial, Tokyo Metropolitan Children’s Medical Center, 2-8-9 Musashidai, Fuchu, Tokyo 183-8561 Japan; 4 Department of Pediatric Nephrology, Aichi Children’s Health and Medical Center, 1-2 Osakata Morioka-cho, Obu, Aichi 474-8710 Japan; 5 Present Address: Japanese Red Cross Toyota College of Nursing, Clinical Medicine, 12-33 Nanamagari, Hakusan-cho, Toyota, Aichi 471-8565 Japan

**Keywords:** Children’s Perceived Competence Scale, Reliability, Validity, Elementary school, Junior high school, Children, Adolescents

## Abstract

**Background:**

It is important for children to maintain high self-perceived competence and self-esteem, and there are few measures to evaluate them through elementary to junior high school days in Japan. To evaluate psychometric properties of the Children’s Perceived Competence Scale (CPCS).

**Methods:**

Data were collected from 697 elementary school and 956 junior high school students. Some of these students completed measures for construct validity, whereas others repeated the CPCS.

**Results:**

The results demonstrated the three-factor structure of the CPCS: cognitive (nine items), social (eight items) and physical (nine items). Factorial invariance was confirmed between elementary and junior high school students, as well as between boys and girls. Construct validity was excellent. Scores on the cognitive, physical and general self-worth domains declined with increasing age. Boys scored significantly higher than girls on physical and general self-worth domains.

**Conclusions:**

The CPCS is a valid and reliable measure of perceived competence in Japanese children aged 6–15 years. The CPCS may be applied to students from elementary through junior high school days as a measure of self-perceived and psychological state in Japan.

## Background

Positive self-esteem has been found to protect children and adolescents from distress and despondency, enabling them to cope with difficult and stressful life situations [1]. A positive sense of self is regarded as central to the adaptive function of individuals [2], with self-perceived competence being an indispensable emotion and the central issue for children and adolescents in the educational, psychological and medical fields. At least one study has revealed the positive role of self-esteem in emotional states (depression and anxiety) in adolescents aged 13–18 [3]. Maintaining positive or high self-esteem is a very important aspect in pediatric medicine, especially for children with chronic diseases [4]. Self-perception or quality of life varies with chronic diseases, as scores in adolescents with autism spectrum disorders were significantly lower than those in controls and diabetic adolescents, especially in the factors friendships, leisure time, and affective and sexual relationships [5]. Although the most appropriate method to determine a child’s viewpoint by psychological assessment is currently regarded to be a semi-structured interview [6], clinicians and medical workers in the pediatric field have long sought a useful measure of self-esteem as an objective and supplementary yardstick.

Self or self-esteem has been described using different terms, including self-worth, self-perception, self-representation, self-evaluation, and self-perceived competence. The term “perceived competence” was selected based on theories of competence and intrinsic motivation [7–9]. Until the 1970s, a single score approach was utilized to evaluate children’s general sense of self or self-esteem [10–12]. In the 1980s, the single score approach was found to mask many important evaluative distinctions regarding children’s competence or adequacy in various domains of their lives. Abstract reasoning was reported to begin at around 12 years of age, with abstraction of self traits also beginning in early adolescence (age 11–13 years) [7, 13, 14]. These findings, that pre-adolescents tend to express elaborate taxonomic attributes and focus on specific competencies, led to the development of a multi-dimensional or domain-specific approach to child development [15–17]. This resulted in the formulation of two determinants, with success in domains deemed important determined by self and the reflected appraisals of others [18, 19].

The Children’s Perceived Competence Scale (CPCS) in this study is a revised Japanese version of the Perceived Competence Scale for Children (PCSC) developed by Harter which is a well-known worldwide multi-dimensional questionnaires [20]. Although several questionnaires in Japanese are used to assess self-perceived competence, quality of life, and self-esteem, few of these measures show good reliability and validity for children of both elementary and junior high school age [21, 22].

PCSC first developed in 1982 [17, 23], consists of seven items in each of four domains (cognitive, social, physical, and general self-worth) for children aged 8–15 years. Each of these 28 items utilized two-step dichotomous scales. Although the Japanese version of the PCSC was shown to have good validity and reliability [24], it was revised in 1992 because its two-step dichotomous scale is difficult to apply to elementary school children in a very large survey. This led to the development of a revised version of the Japanese PCSC version, CPCS [25]. This scale consists of 10 items in each of the four domains (cognitive, social, physical, and general self-worth). Although the contents of the PCSC and CPCS are similar, the CPCS is easier to understand due to its simplicity and clarity of sentences and contents and its four-point scale system. Harter developed various revised or new versions of the PCSC, including the Pictorial Scale of Perceived Competence and Acceptance for young children aged 4–7 years in 1983, the self perception profile for children (SPPC) for children aged 8–13 years in 1985 and 2012, the self-perception profile for adolescents for children and adolescents aged 14–18 years in 1988 and 2012 and others [26, 27].

The SPPC consists of the three subscales and general self-worth domain which are in CPCS, along with two additional subscales, physical appearance and behavioral conduct [20, 28–30].

Although the validity and reliability of the Japanese version of the SPPC has been evaluated [31–33], exploratory factor analysis by three different studies each reported different results and low factor loadings on many items, especially in behavioral conduct. Also only one of the three studies reported confirmatory factor analysis recommending different models for students in elementary and junior high school and the inclusion of only three of the six items in the behavioral conduct domain [31]. Therefore, we abandoned the idea of adopting SPPC in favor of the CPCS, the revised Japanese version of the PCSC. General self-worth scores on the CPCS are closely associated with overall self-esteem. Although the subscale reliability of the Japanese version of the CPCS was assessed using Cronbach’s alpha, its validity was only partially evaluated [24, 25]. Also our previous study of CPCS scores in elementary school children found scores decreased significantly as children progressed from school grade four to school grade five (approximately age 10–11 years) [34]. This period, namely the beginning of adolescence, is very important for paying attention to the self-esteem of children, and it is desirable to follow up the situation during adolescence. In the present study, continuously applying the CPCS to students in elementary school through junior high school in Japan, we attempted to reevaluate the validity and reliability of the CPCS.

## Methods

### Participants

This study involved 698 students, 350 boys and 348 girls, enrolled in three elementary schools and 992 students, 524 boys and 468 girls, enrolled in four junior high schools in Aichi prefecture, Japan, between September and December 2014. Aichi has the fourth largest population of the 47 Prefectures in Japan, with a population of about 7.5 million. To sample a representative cross-section of the target-age population, three elementary schools were selected, one located in old downtown, one in a rural seacoast town and one in the largest city. Similarly, four junior high schools were selected, one located in old downtown, one in a rural seacoast town and two in the largest city.

### Measures

#### CPCS

The CPCS is a Japanese questionnaire derived from the PCSC. This instrument was designed to measure children’s self-perception of competence across the cognitive (scholastic) (10 items, e.g., “Are you among the best in your class at studying?”), social (peer relationship) (10 items, e.g., “Do you have many friends?”) and physical (athletic) (10 items, e.g., “Are you good at sports?”) domains, as well as general self-worth (self-esteem) (10 items, e.g., “Do you have self-confidence?”). General self-worth is regarded as self-esteem, a concept different from that in the other three competence domains. CPCS subscale scores indicate a child’s perceived self-competence in each domain. Each item was scored on a four-point scale: true (4), relatively true (3), relatively false (2), and false (1). Therefore, the score on each domain ranged from 10 to 40, with higher scores indicating greater perceived self-competence. The CPCS was administered in a classroom setting and completed by the students themselves.

#### Kid-KINDL^R^ and Kiddo-KINDL^R^

These tests are self-reported instruments for assessing generic health-related quality of life in children aged from 6 to 12 and 13 to 18 years old [35]. The questionnaire consists of 24 items addressing six subscales: physical well-being, emotional well-being, self-esteem, family, friends, and school life. Three versions of the KINDL are available, the kiddy-KINDL^R^ for children aged 4–6 years old, the Kid-KINDL^R^ for children aged 7–13 years and the Kiddo-KINDL^R^ for children aged 14–17 years. Japanese versions of the Kid-KINDL and Kiddo-KINDL were developed for elementary school [36] and junior high school [22] students. Self-esteem, friends and school life in the Kid-KINDL^R^ correspond to general self-worth, social, and cognitive domains, respectively, in the CPCS.

#### Rosenberg Self Esteem Scale

The Rosenberg Self Esteem Scale [37] (RSES), comprising 10 items, is the most commonly used instrument for measuring self-esteem in adolescents and adults. A Japanese version of the RSES [38] was administered to junior high school students in this study.

#### The grade of physical performance test

Japanese students undergo nationwide physical performance tests once a year. The grade of physical performance on these tests, graded as excellent (81–100 percentile), average (20–80 percentile), and poor (0–20 percentile), was provided by teachers at each school.

#### Chronic disease

Each school nurse answered yes or no about presence of chronic diseases based on the health information report of each student. Chronic diseases were defined as mental and physical illnesses requiring regular hospital stay or visits, or requiring special attention by teachers.

### Procedure

Of the 698 elementary school students invited to participate, 697 (99.9%), 349 boys and 348 girls, retuned questionnaires. Similarly, of the 992 junior high school students invited to participate, 956 (96.4%), 498 boys and 458 girls, returned questionnaires. All completed the CPCS. To validate the CPCS, the Kid-KINDL^R^ was also administered to 450 elementary school students and the Kiddo-KINDLE^R^ and RSES were administered to 305 junior high school students. To assess the test–retest reliability of the CPCS, this test was administered twice to 336 students, at intervals of 7–14 days. Students selected for testing by the Kid-KINDL, Kiddo-KINDL and RSES and those selected for retesting with the CPCS were chosen by the principal of each school.

### Statistical analysis

The sample was randomly divided into two groups, with the first group undergoing exploratory factor analysis (EFA) for the CPCS items. After determining the means, standard deviations, and skewness of the CPCS items, factors were extracted based on the maximum-likelihood method, with the number of factors determined by the scree plot method. Following Promax rotation, a diagonal rotation method, CPCS items belonging to three subscales, Cognitive, Social, and Physical, were entered into EFA. Because the General self-worth subscale was regarded as qualitatively different from the other specific domains of competence, the General self-worth subscale scores were regarded as a measure of construct validity.

The factor structure extracted through the EFA was cross-validated by a series of confirmatory factor analyses (CFAs) using the second group. Factor models were revised by adding correlations between error variables according to modification indices. These were added only to error variables belonging to the same factor. The fit of each model to the data was examined using Chi squared (CMIN), comparative fit index (CFI), and root mean square error of approximation (RMSEA). According to conventional criteria, a good fit would be indicated by CMIN/*df *< 2, CFI > .97, and RMSEA < .05, and an acceptable fit by CMIN/*df* < 3, CFI > .95, and RMSEA < .08 [39].

After determining factor structure, measurement invariance between elementary and junior high school students as well as between boys and girls was determined by weak factorial variance, in which loading of each factor was the same between groups. If weak factor invariance was not confirmed, the path and indicator with the greatest critical ratio for difference in factor loading were deleted, and the examination was repeated until weak factor invariance was obtained. After determining the factor structure of weak factorial invariance between elementary and junior high school students, it was subjected to examination of weak factorial invariance between boys and girls. Finally, a factor structure with weak factorial invariance between elementary and junior high school students and between boys and girls was obtained. The above treatment resulted in the creation of subscales of the CPCS, with each subscale calculated by adding scores of items belonging to each factor. The correlations between the scores of these CPCS subscales and demographic variables, Kid-KINDL (Kiddo-KINDL) scores and RSES scores were assessed.

The reliability of each CPCS subscale was examined by determining its internal consistency (Cronbach’s alpha coefficient) and test–retest reliability (intraclass correlation coefficient: ICC). Finally, the distribution of CPCS subscale scores by age (grade) was determined separately in boys and girls.

Missing values were determined by adopting list-wise deletion for EFAs and CFAs. Multiple imputations were used when analysing all data for validation of the CPCS subscales. Little’s MCAR test showed that the null hypothesis (the data were missing completely at random) was rejected (Chi squared = 4241.5, *df* = 3641, *P* < .001). All statistical analyses were performed using SPSS version 20.

## Results

### CPCS: item scores and gender difference

The population of elementary and junior high school students was divided randomly into two groups. Evaluation of the first group (*n* = 797) showed that the distribution of all CPCS items was relatively non-skewed (Table 1). Boys and girls differed mainly in the physical and general self-worth subscales. Boys scored significantly higher than girls on physical items such as “good at sports” and “interested in trying new sports” and significantly lower on items such as “prefer watching rather than participating in sports” and “dislike PE”. Boys also scored significantly higher on general self-worth items such as “have self-confidence”, “have a lot of things to be proud of” and “have confidence in one’s own opinions” and significantly lower on items such as “not a useful person” than girls.

**Table 1:** Exploratory factor analysis of the CPCS (N = 797)

Item no.	Contents	M (SD)				Skewness
		Total	Boys	Girls	Gender difference	
	Cognitive					
1	Among the best at studying	2.44 (1.00)	2.45 (1.01)	2.43 (1.00)	t (791) = .3	− .0
5	Poor at studying (R)	2.41 (1.11)	2.42 (1.13)	2.40 (1.09)	t (793) = .2	.1
9	You are one of the cleverest	2.20 (1.02)	2.19 (1.02)	2.21 (1.02)	t (794) = .2	.2
13	Your grades are bad (R)	2.51 (1.09)	2.45 (1.13)	2.57 (1.05)	t (791.9) = 1.5	− .1
17	Finish your homework quickly	2.71 (1.07)	2.73 (1.12)	2.68 (1.02)	t (791.2) = .6	− .3
21	Understand school lessons well	2.98 (.92)	2.97 (1.00)	2.99 (.85)	t (788.3) = .3	− .6
25	Unable to answer questions (R)	2.82 (.98)	2.77 (1.04)	2.88 (.91)	t (789.0) = 1.6	− .5
29	Attempt challenging problems	2.82 (1.07)	2.86 (1.09)	2.78 (1.05)	t (792) = 1.1	− .4
33	Get good scores in exams	2.32 (1.02)	2.33 (1.03)	2.31 (1.03)	t (794) = .4	.1
37	Express your opinions in class	2.41 (1.11)	2.54 (1.12)	2.27 (1.09)	t (795) = 3.5	.1
	Social					
2	Have many friends	3.36 (.83)	3.39 (.83)	3.31 (.82)	t (793) = 1.3	− 1.2
6	You are popular in your class	2.04 (.90)	2.11 (.94)	1.96 (.86)	t (794.6) = 2.4*	.4
10	Teased by your friends? (R)	3.32 (.94)	3.26 (1.00)	3.38 (.89)	t (790.8) = 1.8	− 1.2
14	Nobody worries when you are absent (R)	2.72 (1.00)	2.72 (1.00)	2.72 (1.00)	t (794) = .1	− .3
18	Easy to make friends	2.69 (1.11)	2.74 (1.09)	2.63 (1.13)	t (793) = 1.4	− .3
22	Friends invite you to play	3.08 (1.00)	3.09 (1.00)	3.07 (.95)	t (795) = .4	− .8
26	You are the hub of the class	2.12 (1.00)	2.16 (.98)	2.08 (.93)	t (791) = 1.3	.4
30	You are liked by your friends	2.65 (1.00)	2.65 (.97)	2.65 (.94)	t (792) = .0	− .4
34	People do not pay much attention to you (R)	3.25 (.86)	3.20 (.89)	3.31 (.83)	t (790) = 1.9	− 1.1
38	People will feel sad if change school	2.82 (1.00)	2.76 (1.02)	2.88 (.98)	t (791.8) = 1.7	− .5
	Physical					
3	Good at sports	2.81 (1.06)	2.95 (1.01)	2.65 (1.09)	t (780.0) = 4.0***	− .4
7	Confidence in new sports	2.43 (1.08)	2.53 (1.07)	2.32 (1.08)	t (793) = 2.8**	.1
11	Chosen for the sports meets	2.12 (1.15)	2.17 (1.18)	2.07 (1.11)	t (793.0) = 1.1	.5
15	Prefer watching rather than participating in sports (R)	2.94 (1.20)	3.05 (1.19)	2.82 (1.21)	t (785.4) = 2.7**	− .6
19	Interested in trying new sports	2.83 (1.16)	2.90 (1.15)	2.75 (1.17)	t (794) = 1.8	− .4
23	Dislike PE (R)	3.28 (1.00)	3.43 (.91)	3.13 (1.02)	t (771.9) = 4.3***	− 1.2
27	Poor at sports	2.94 (1.16)	3.12 (1.09)	2.76 (1.19)	t (776.3) = 4.5***	− .6
31	Not lose to friends at sports	2.33 (1.09)	2.43 (1.08)	2.23 (1.09)	t (793) = 2.7**	.2
35	Amongst the best at sports	2.53 (1.05)	2.60 (1.15)	2.33 (1.33)	t (792) = 3.3**	− 1.4
39	Prefer not to be seen by others when you do sports (R)	2.91 (1.14)	3.02 (1.13)	2.79 (1.14)	t (794) = 2.9**	− .6
	General self-worth					
4	Have self-confidence	2.48 (1.00)	2.54 (.89)	2.40 (1.00)	t (791) = 2.0*	.0
8	Do things better than others	2.34 (.92)	2.42 (.92)	2.25 (.92)	t (792) = 2.5*	.1
12	Have a lot of things to be proud of	2.20 (1.00)	2.29 (1.04)	2.10 (.94)	t (793.0) = 2.7**	.4
16	Does not go well whatever you do (R)	2.89 (1.01)	2.97 (1.03)	2.82 (1.00)	t (791) = 2.1*	− .6
20	Satisfied with the way you are now	2.39 (1.09)	2.45 (1.10)	2.34 (1.07)	t (795) = 1.4	.1
24	Will surely become a great person	1.84 (.94)	1.94 (1.00)	1.73 (.89)	t (791) = 3.1**	.9
28	Not a useful person (R)	2.63 (1.00)	2.68 (.88)	2.58 (.95)	t (795) = 1.4	− .3
32	Have confidence in your own opinion	2.51 (1.06)	2.60 (1.07)	2.42 (1.05)	t (788) = 2.4*	.0
36	Have few good points (R)	2.53 (1.06)	2.54 (1.09)	2.52 (.99)	t (792.8) = .2	− .1
40	Worried about whether or not you fail (R)	2.37 (1.15)	2.52 (1.17)	2.22 (1.10)	t (794.0) = 3.7***	.2

*R* reversed

* *P* < .05; ** *P* < .01; *** *P* < .001

### Factor structure of the CPCS

The scree plot for EFA on data from the first group (*n* = 797) suggested a three-factor structure. The first factor included CPCS items belonging to the physical domain; the second factor included items belonging to the cognitive domain; and the third factor included items belonging to the social domain (Table 2).

**Table 2:** Exploratory factor analysis of the CPCS (N = 797)

Item no.	Contents	Factor		
		1	2	3
	Cognitive			
1	Among the best at studying	.03	*.83*	− .11
5	Poor at studying (R)	.03	*.71*	− .14
9	One of the cleverest	− .02	*.86*	− .04
13	Your grades are bad (R)	− .12	*.74*	− .00
17	Finish your homework quickly	.04	*.46*	.02
21	Understand school lessons well	− .04	*.64*	.02
25	Unable to answer teachers’ questions (R)	− .05	*.42*	.14
29	Attempt challenging problems	.08	*.58*	.05
33	Get good scores in exams	− .04	*.84*	− .03
37	Express your opinions in class	.08	*.44*	.18
	Social			
2	Have many friends	.21	.02	*.51*
6	Popular in your class	.17	.13	*.47*
10	Teased by your friends (R)	− .11	− .16	*.41*
14	Nobody worries when you are absent (R)	− .10	.11	*.59*
18	Easy to make friends	.20	− .01	*.32*
22	Friends invite you to play	.09	− .11	*.52*
26	The hub of the class	.07	.17	*.36*
30	Liked by your friends	− .05	.02	*.65*
34	People do not pay much attention to you (R)	− .07	− .07	*.62*
38	People will feel sad if change school	− .01	.09	*.69*
	Physical			
3	Good at sports	*.93*	− .03	− .09
7	Confidence in new sports	*.77*	.08	− .04
11	Chosen for the sports meets	*.61*	.04	.02
15	Prefer watching rather than participating in sports (R)	*.60*	− .02	.01
19	Interested in trying new sports	*.66*	− .04	.07
23	Dislike PE (R)	*.60*	− .05	.04
27	Poor at sports	*.87*	− .11	− .08
31	Not lose to friends at sports	*.72*	.09	− .01
35	Amongst the best at sports	*.85*	.06	− .07
39	Prefer not to be seen by others when you do sports (R)	*.38*	− .12	− .19

Factor loadings of .30 or more are in italics

**Fig. 1:**
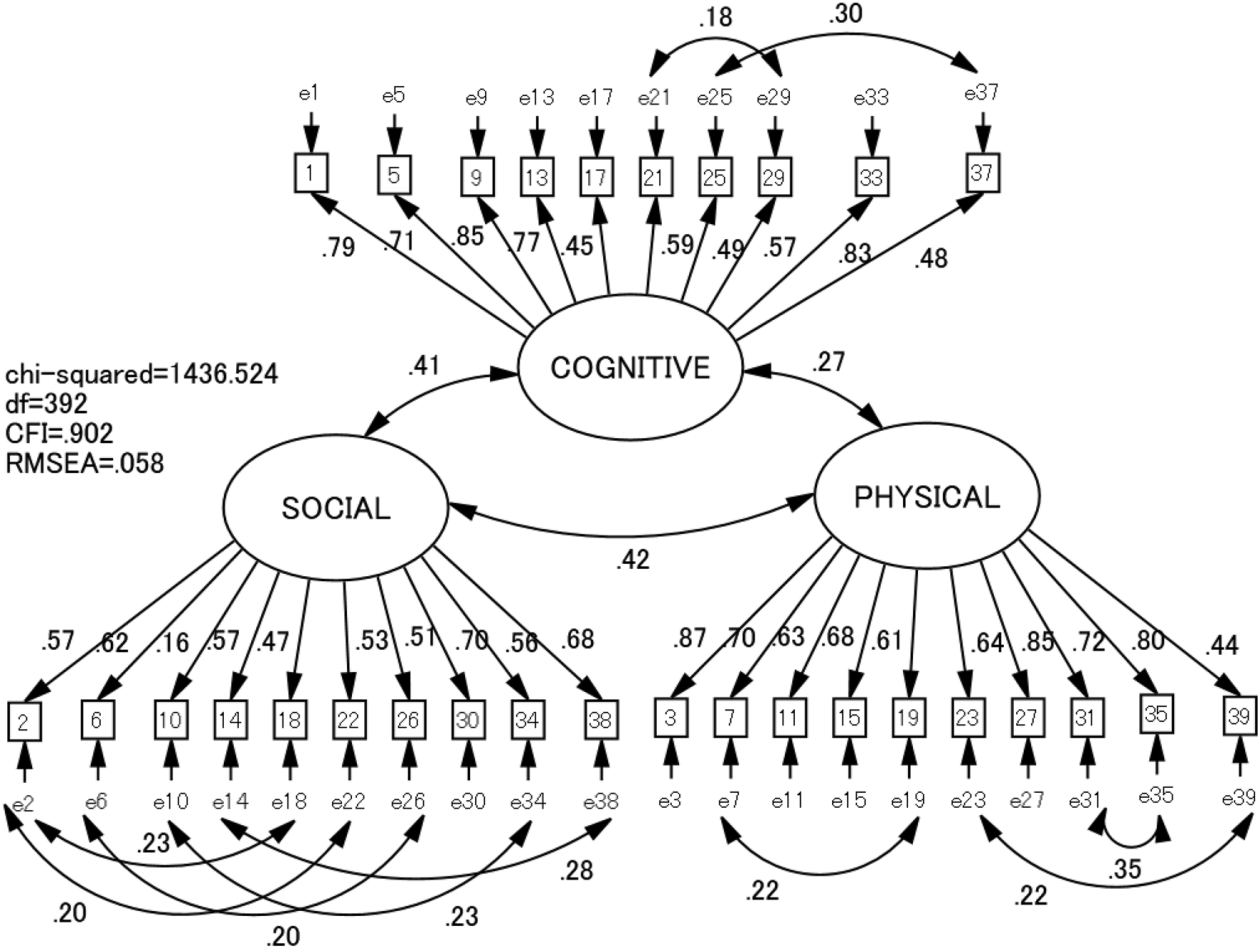
Revised model of confirmatory factor analysis and goodness-of-fit indices

Cross-validation of the factor structure of the CPCS was examined by a CFA on the second group of students (*n* = 792). The original three-factor model without correlations between error variables showed non-acceptable goodness-of-fit: Chi squared/*df* = 4.7, CFI = .861, and RMSEA = .068. The addition of inter-error correlations according to the modification indices resulted in a revised model (Fig. 1), which showed better goodness-of-fit indices: Chi squared/*df* = 3.7, CFI = .902, and RMSEA = .058.

**Fig. 2:**
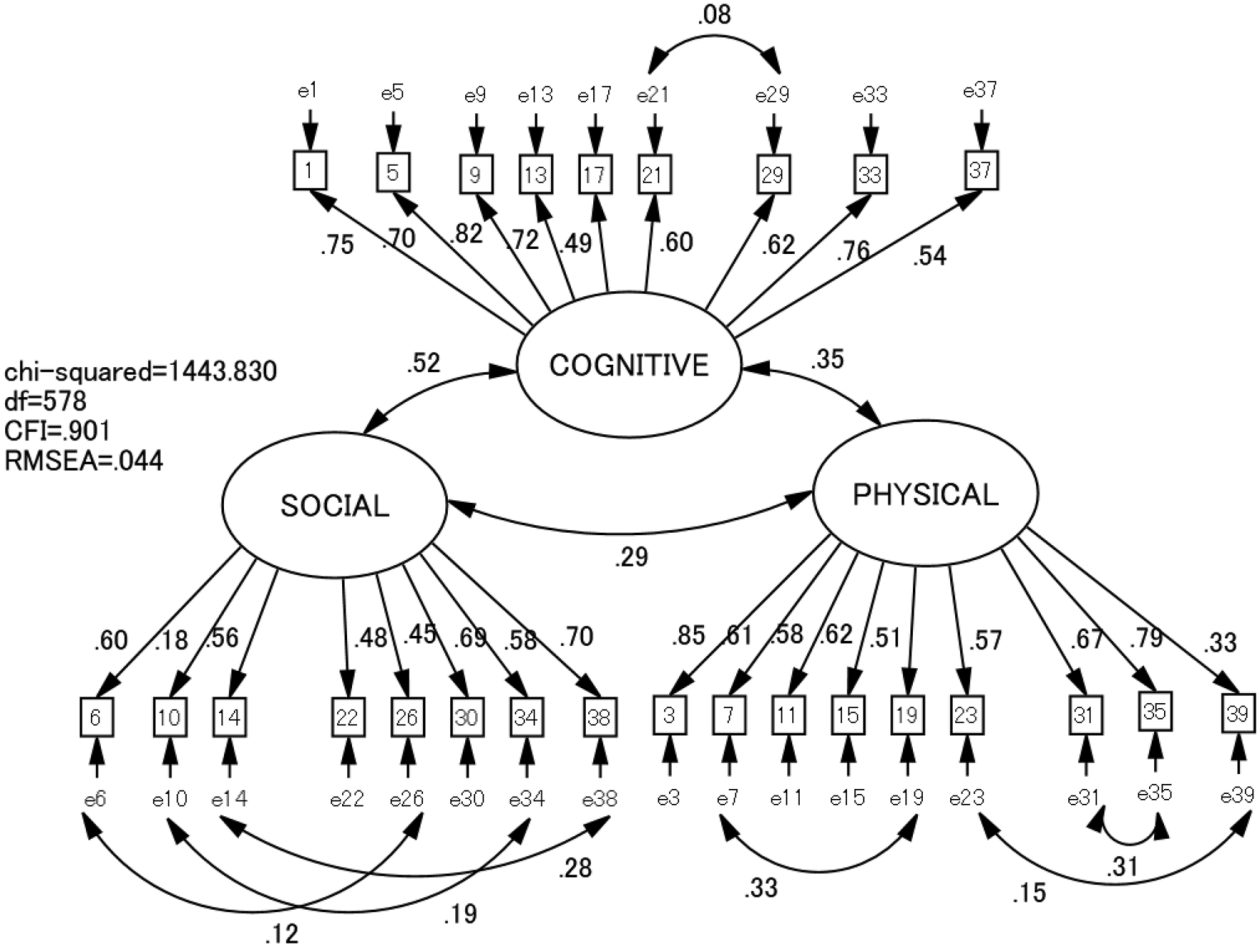
Final model of Children’s Perceived Competence Scale (26 items) and goodness-of-fit indices

We also examined whether the 3-factor structure showed factorial invariance between elementary and junior high school students as well as between boys and girls. Multigroup analysis revealed a difference between elementary and junior high school students (increase of Chi squared = 63.684, *df* = 27, *P* < .001). Because weak measurement invariance was refuted, we searched critical ratios for differences between parameters in the two groups. We individually deleted parameters (factor loading) with the highest critical ratios until weak factor invariance appeared. This was obtained by deleting four CPCS items (items 2, 18, 25, and 27), yielding Chi squared/*df* = 2.4, CFI = .901, and RMSEA = .044 (Fig. 2).

Using this three-factor model with 26 items, we examined whether boys and girls differed in factor structure. Compared with the model without constraining parameters, the model that constrained factor loadings between boys and girls did not differ significantly in increased Chi squared (22.094, *df* = 23, *P* = .515). Weak factorial invariance between the two genders was confirmed by this model. Hence this final model showed weak factorial invariance both between elementary and junior high school students and between boys and girls. The first factor with nine items reflected physical domain, the second factor with nine items reflected cognitive domain, and the third factor with eight items reflected social domain.

The CPCS is usually used as a measure in children of grade 3 and higher. It is not usually administered to children in grades 1 and 2. Therefore, we repeated the test of factorial invariance between children in grades 1–2 and those in grades 3 and higher. The model of restricted parameters for all factor loadings did not exceed the model of free parameters for factor loadings in terms of Chi squared (32.065, *df* = 23, P = .099). Hence weak factorial invariance was confirmed between children in grades 1–2 and those in grades 3–9.

### Concurrent and construct validity of the CPCS

To examine the concurrent and construct validity of the three subscales derived from the above EFA and CFA results, we assessed the 755 students administered the Kid-KINDL and Kiddo-KINDL instruments. We constructed three subscales by adding the scores of items belonging to each factor: cognitive, social and physical. All three subscale scores of the CPCS correlated significantly with general self-worth score (Table 3). In addition, the scores of all six Kid-KINDL and Kiddo-KINDL subscales correlated with the scores of their equivalent CPCS subscales. In the junior high school students, the scores on the RSES correlated with the scores of all three CPCS subscales. Therefore, the scores on the CPCS subscales assessed similar aspects of perception measured by the other scales.

**Table 3:** Correlations of the CPCS subscales with other variables and the comparison of the scores by gender and chronic diseases (N = 755)

	CPCS subscales		
	Cognitive	Social	Physical
CPCS			
General self-worth (r)	.62***	.63***	.52***
Kid-KINDL^R^/Kiddo-KINDL^R^			
Physical health (r)	.26***	.29***	.23***
Emotional well-being (r)	.28***	.58***	.26***
Self-esteem (r)	.55***	.56***	.46***
Family (r)	.29***	.35***	.17***
Friends (r)	.30***	.61***	.32***
School life (r)	.60***	.34***	.27***
RSES (N = 305) (r)	.51***	.62***	.44***
Grade (r)	− .30***	.00	− .23***
Physical performance test (r)	− .10 ***	− .09***	.39***
Gender			
Boys (n = 385) (Mean (SE))	22.9 (.2)	21.9 (.2)	25.3 (.2)
Girls (n = 370) (Mean (SE))	22.6 (.2)	22.9 (.2)	23.2 (.3)
t test	− 1.0 NS	− .6 NS	6.2**
Chronic disease			
None (n = 619) (Mean (SE))	22.7 (.2)	22.0 (.1)	24.3 (1.9)
Present (n = 136) (Mean (SE))	23.3 (.4)	21.6 (.3)	23.9 (.4)
t test	− 1.4 NS	1.2 NS	.9 NS

* *P* < .05; ** *P* < .01; *** *P* < .001; () indicates standard error

The scores on the Social subscale did not correlate with the student’s grades, whereas the scores on the Cognitive (*r* = − .30) and Physical (*r* = − .23) subscales were significantly negatively correlated with grade (Table 3). The Physical performance test scores were moderately correlated with Physical subscale. The comparison of the scores by gender and chronic diseases also indicated (Table 3). Boys showed significantly higher scores in Physical subscale than girls and three CPCS subscale scores did not differ between students with and without chronic diseases.

### Reliability of the CPCS

Cronbach’s alpha coefficients were, .87, .79, and .88 for the cognitive, social, and physical subscales, respectively (Table 4). These were virtually the same when calculated for elementary and junior high school students separately and for boys and girls separately.

**Table 4:** Internal consistency and test–retest reliability of each subscale in CPCS

	CPCS subscales		
	Cognitive	Social	Physical
Internal consistency			
Total (N = 755)	.87	.79	.88
Elementary school (n = 450)	.85	.77	.86
Junior high school (n = 305)	.89	.83	.91
Boys (n = 385)	.88	.80	.86
Girls (n = 370)	.86	.79	.90
Test–retest reliability (ICC)			
Total (n = 431)	.89***	.81***	.90***
Elementary school (n = 336)	.88***	.80***	.89***
Junior high school (n = 95)	.92***	.85***	.88***
Boys (n = 215)	.89***	.83***	.89***
Girls (n = 216)	.89***	.78***	.91***

* *P* < .05; ** *P* < .01; *** *P* < .001

Test–retest reliabilities were, .89, .81, and .90 for cognitive, social, and physical subscales, respectively. These were virtually the same when calculated for elementary and junior high school students separately and for boys and girls separately.

### Age distribution of the CPCS subscale scores

Because the scores of the physical subscale differed in boys and girls, we examined the distribution of scores of the CPCS subscales over age (grade) in boys and girls separately (Table 5).

**Table 5:** Means and standard errors in each subscale of CPCS

	n	CPCS subscales							
		Cognitive		Social		Physical		General	
	Boys:girls	Boys	Girls	Boys	Girls	Boys	Girls	Boys	Girls
1	27:34	28.2 (1.1)	28.4 (.8)	22.6 (1.0)	23.1 (.7)	28.1 (.9)	28.2 (1.1)	28.9 (1.5)	30.0 (.7)
2	28:41	24.4 (1.2)	26.0 (.9)	19.2 (1.2)	21.8 (1.0)	26.9 (1.2)	27.2 (1.0)	24.9 (1.3)	25.4 (1.1)
3	27:36	26.5 (1.2)	23.6 (1.0)	23.5 (1.0)	22.2 (.7)	30.0 (.9)	26.8 (1.1)	30.0 (1.4)	25.2 (.9)
4	47:26	23.5 (.9)	24.3 (1.1)	20.6 (.6)	21.3 (1.0)	25.6 (1.0)	23.5 (1.5)	24.0 (1.0)	23.4 (1.5)
5	44:31	22.7 (1.0)	22.2 (1.1)	20.1 (.8)	21.1 (.8)	25.0 (1.0)	21.8 (1.5)	22.6 (1.1)	22.1 (1.2)
6	52:57	24.7 (.9)	23.4 (.7)	23.4 (.6)	22.1 (.7)	26.5 (.8)	22.7 (.9)	26.0 (.8)	22.8 (.9)
7	49:51	22.1 (1.0)	21.4 (.9)	22.2 (.7)	21.4 (.7)	25.3 (1.0)	22.6 (1.2)	24.0 (1.1)	22.5 (.9)
8	73:58	20.8 (.8)	22.1 (.8)	22.0 (.6)	Grade	24.0 (.8)	22.8 (1.0)	23.6 (.8)	22.7 (.7)
9	38:36	20.6 (1.1)	20.6 (1.0)	21.3 (.6)	21.6 (.6)	23.4 (1.1)	22.9 (1.0)	22.9 (1.2)	21.8 (.8)
	F	5.66***	6.58***	3.40**	.58 ns	3.54**	4.45***	4.52***	6.43***
Post hoc comparison		1 > 5, 7–9 3 > 8, 9 6 > 8	1 > 3, 5, 6–9 2 > 7–9	2 < 6 5 < 6	–	3 > 5, 8, 9	1 > 5, 6–9 2 > 6, 7	1 > 5, 8, 9 3 > 4, 5, 7–9	1 > 2–9

() Standard error. * *P* < .05; ** *P* < .01; *** *P *< .001

All three CPCS subscale scores differed among the nine grades in boys, whereas both Cognitive and Physical subscale scores differed over these grades in girls. Post-hoc comparison (Tukey) showed that both boys and girls scored higher in lower than in higher grades, except for social subscale scores in boys.

## Discussion

### Validity and reliability

In the present study, we reevaluated the validity and reliability of the CPCS. All the CPCS items, except for General self-worth, were used for an EFA. The three factors extracted from the EFA were in agreement with theoretic expectations. Our final model showed acceptable fit in a CFA. One strength of our study was its assessment of factor invariance. We identified the factor structure that was invariant between age groups and between genders. In most investigations examining different populations, the robustness of the measurement is of great concern. Researchers wish to ensure that the questionnaire items used in measurements did not greatly alter their relationship to the latent variables across populations with different attributes. Any observed difference would make the interpretation of the results derived from that measure difficult. We identified weak factorial invariance between age groups as well as between genders after deleting a few CPCS items. This means that a simple summation of items may distort the results of the data.

The validity of the three subscales derived from the EFA, CFA, and deletion of a few items that reduced factor invariance was examined for its relationship with General self-worth, as well as with Kid KINDLE, Kiddo KINDLE, and RSES scores. All results were as expected.

Reliability, as determined by internal consistency and test–retest formats, was also excellent. This is consistent with a report showing that Chronbach’s alpha ranged between .75 and .87 [40].

### Gender differences

Evaluation of students in grades 3–9 showed that boys consistently showed significantly higher scores than girls on the physical (athletic) subscale [17]. In addition, many studies have reported that boys score higher than girls on measures of self-esteem [22, 35, 41]. Our findings, that boys scored higher than girls on Physical and general self-worth subscales, were compatible with these previous reports. The higher Physical scores in boys than in girls, especially those in junior high school, may be due to gender differences in body composition among junior high school students varies, with boys having more muscle mass and girls having more fat mass [42]. Gender differences on the general self-worth subscale may be explained, at least in part, by girls expressing more self-conscious emotions, like shame and guilt, than boys [43, 44]. Moreover, the lower scores on Physical subscales in girls may also contribute to their lower scores on Gen eral self-worth.

### Chronic diseases

In our study the three CPCS subscale scores did not differ between students with and without chronic diseases. Thus, all students were included in our analyses. Most of the students with chronic diseases in our study had mild allergic diseases, including allergic rhinitis and atopic dermatitis, allowing regular school attendance. The school nurse-provided information may have contributed to this result.

### Grade differences

Several studies using self-administered questionnaires have assessed self-perception or self-esteem in students from elementary through junior high school. A study using Kid-KINDL and Kiddo-KINDL in Japanese elementary and junior high school students showed that school life (scholastic) and self-esteem domains decreased markedly with age, whereas friends (social) domain did not [21, 22]. In contrast, another study found no marked changes with age [17]. Scores on the Self Description Questionnaire I, which measures seven specific self-concept factors (physical ability, physical appearance, peer relationship, parent relationship, reading, mathematics and general school), declined linearly with age in all subscales in students in grades 2–9 [45]. We observed age-related reductions in cognitive, physical, and general self-worth subscale scores, with some fluctuation, but no consistent pattern, in social subscale scores.

Adolescents aged 11–16 years gradually evaluate their attributes via interactions with others and with regarded to different roles and relational contexts. They can recognize positive and negative attributes simultaneously, inter-coordinate their traits into single abstractions and form links between single abstractions. This process of preoccupation with others’ thoughts of the self and cognition about contradictory characteristics of the self tends to lead to confusion and inaccuracies [14]. This view of adolescence provides an appropriate guide to understanding its description as “a period of crisis” [46].

### Limitations

The present study has limitations. First, the population studied was selected from schools in Aichi prefecture, one of 47 prefectures in Japan. Although we tried to balance socio-economic variables by selecting elementary and junior high schools differing in academic level, local economy, industry, and population, a nationwide survey may yield different reference values. Generalization of our results to the entire Japanese population requires an expansion of the number of locations. Second, we used the CPCS, a Japanese revised version of the PCSC. Thus, it may be difficult to compare our data directly with data obtained using the PCSC in other countries. Studies have reported that self-esteem is much lower in Japanese children than in children in other countries. For example, almost all scores on QOL subscales, including self-esteem in Kid-KINDL, in elementary school children were lower in Japan than in Finland [47]. Scores on all subscales of the Self-Description Questionnaire, including general self-esteem, were found to be higher in elementary school children in the USA than in Japan [48]. Although the result of CPCS in our study cannot be used for research on international comparison, this study may provide insights on how to evaluate and create measures of self-esteem for children and adolescents.

Third, the validity of the information on presence of disease might be weak as some students may have had more slight symptoms than others and as the students with chronic disease were defined by a yes or no report regarding their health.

Fourth, the students selected by principals for test–retest reliability might have undergone selection bias. School events and education curriculum prevented the selection of a randomized sample.

## Conclusion

This study reevaluated the validity and reliability of the CPCS. The results indicate that the CPCS can be applied to children and adolescents attending elementary and junior high schools regardless of suffering from chronic diseases. The CPCS may be a useful measure to evaluate the mental state of students from age 6 through 15 years not only in a medical field but in an educational or social welfare field. Although the general self-worth subscale is not included in the CPCS model in our study, its three subscales (cognitive, social, physical) may effectively measure important aspects of self-esteem in children and adolescents.
